# 下调TRAF6表达对肺癌细胞株恶性生物学行为的影响

**DOI:** 10.3779/j.issn.1009-3419.2015.11.01

**Published:** 2015-11-20

**Authors:** 根 林, 传钟 黄, 光建 苏, 卉华 胡, 海鹏 徐, 诚 黄

**Affiliations:** 350014 福州，福建医科大学教学医院，福建省肿瘤医院胸部肿瘤内科 Department of Medical Oncology, Fujian Provincial Cancer Hospital, Fujian Medical University Teaching Hospital, Fuzhou 350014, China

**Keywords:** 肺肿瘤, 肿瘤坏死因子受体相关因子6, 核因子-κB, 凋亡, 侵袭, Lung neoplasms, Tumor necrosis factor receptor-associated factor 6, Nuclear factor-kappa B, Apoptosis, Invasion

## Abstract

**背景与目的:**

已有的研究提示肿瘤坏死因子受体相关因子6(tumor necrosis factor receptor-associated factor 6, TRAF6)在肺癌中常常扩增，可能扮演癌基因角色，但TRAF6的确切作用尚未充分阐明。本研究探索TRAF6表达对肺癌细胞株的增殖、凋亡、细胞周期、迁移及侵袭能力的影响以及可能作用机制。

**方法:**

选用A549、H1650、SPC-A-1以及Calu-3等四种肺癌细胞株，应用蛋白印迹、qRT-PCR检测其TRAF6蛋白及mRNA表达。SPC-A-1、Calu-3细胞转染TRAF6 siRNA，以EMSA方法检测不同处理组核因子-κB的DNA结合活性，MTS法检测细胞增殖，流式细胞仪PI染色检测细胞凋亡，流式细胞仪进行细胞周期测定，划痕实验及Transwell小室法检测细胞迁移及侵袭能力，并应用蛋白印迹检测泛素化抗体、p65、CD24、CXCR4等蛋白表达。SPC-A-1细胞提取DNA后，应用二代测序法进行全基因组测序。

**结果:**

在四种细胞株中，SPC-A-1和Calu-3细胞TRAF6相对高表达，TRAF6发生自身K63-泛素化，但仅在SPC-A-1细胞中观察到核因子-κB组成性活化。转染TRAF6 siRNA后，SPC-A-1、Calu-3细胞TRAF6表达明显下调，与空白组及对照组相比，下调TRAF6表达可抑制SPC-A-1细胞核因子-κB活性、降低迁移及侵袭能力以及促进细胞凋亡，CD24和CXCR4的表达也明显下调，但对细胞增殖及细胞周期无明显影响。下调TRAF6表达对Calu-3细胞株的核因子-κB活性、细胞增殖、凋亡、细胞周期、迁移及侵袭能力等均无明显影响。未发现SPC-A-1细胞株*TRAF6*基因突变或拷贝数改变。

**结论:**

下调TRAF6表达可抑制SPC-A-1细胞迁移及侵袭能力，促进细胞凋亡，并且TRAF6可能是通过调控核因子-κB-CD24/CXCR4信号通路参与调控肺癌侵袭、细胞凋亡。

## Abstract (en)

**Background and objective:**

It has been proven that tumor necrosis factor receptor-associated factor 6 (TRAF6) was a commonly amplified oncogene in lung cancer. However, the precise role of TRAF6 protein in lung cancer has not been extensively investigated. This study analyzed the effects of TRAF6 on the proliferation, apoptosis, cell cycle, migration, and invasion capability of lung cancer cell lines, as well as the potential molecular mechanisms involved.

**Methods:**

To address the expression of TRAF6 in lung cancer cells, four lung cancer cell lines (A549, H1650, SPC-A-1 and Calu-3) were assayed to determine the expression of TRAF6 protein by Western blot and TRAF6 mRNA via qRT-PCR. Moreover, siRNA targeting TRAF6 was introduced into SPC-A-1 and Calu-3 cells. Nuclear factor-қB (NF-қB) DNA-binding activity, apoptosis rates, cell proliferation, cell cycle, migration, and invasion were determined by electrophoretic mobility shift assay, flow cytometry, MTS assay, flow cytometry, scratch test, and transwell chamber assay, respectively. Western blot analysis was also performed to evaluate the expression of the following proteins through K63-ubiquitination: P65, CD24 and CXCR4. Whole-genome sequencing analysis was conducted using a second-generation sequencer in SPC-A-1 cells.

**Results:**

TRAF6 was highly up-expressed in SPC-A-1 and Calu-3 cell lines than the other two cells, which also showed K63-ubiquitinization in TRAF6. However, constitutive activation of NF-қB was observed only in SPC-A-1 lung cancer cells. Downregulation of TRAF6 suppressed the NF-κB activation, cell migration, and invasion but promoted the cell apoptosis of SPC-A-1 cells. Markedly decreased expression of CD24 and CXCR4 was observed in SPC-A-1 cells transfected by TRAF6 siRNA. Nevertheless, TRAF6 downregulation did not affect the proliferation and cell cycle of SPC-A-1 cells. Additionally, TRAF6 regulation did not affect the proliferation, apoptosis, cell cycle, migration, and invasion of Calu-3 cells. No mutations and no changes in gene copy numbers of TRAF6 were found by whole-exome sequencing of SPC-A-1 cells.

**Conclusion:**

TRAF6 may be involved in cell migration, invasion, and apoptosis of SPC-A-1 cells, possibly through regulating the NF-қB-CD24/CXCR4 pathway.

肿瘤坏死因子受体相关因子6(tumor necrosis factor receptor-associated factor 6, TRAF6)属于肿瘤坏死因子受体相关蛋白家族的一个重要成员，TRAF蛋白家族一共有7个成员，分别是TRAF1-7。TRAFs能直接或间接与多种肿瘤坏死因子和白细胞介素-1(interleukin-1, IL-1)/Toll样受体家族成员结合，介导多种下游信号通路的信号传导，其中包括核因子-қB(nuclear factor-қB, NF-қB)信号通路，从而影响细胞增殖、分化和死亡，并参与多个生物学过程的调控^[1]^。

在几乎所有的NF-қB信号通路中，TRAFs都是关键的信号中间物，其中最主要有TRAF2、TRAF5和TRAF6。TRAF6由530个氨基酸组成，相对分子质量(Mt)约为60, 000，TRAF6在结构上可分为C端的TRAF结构域和N端的激活结构域。TRAF结构域是由含螺旋卷曲结构的TRAF-N结构域和高度保守的TRAF-C结构域组成，主要功能是介导TRAF分子之间形成同源/异源二(多)聚体，并与受体蛋白的TRAF结构域相互作用。TRAF激活结构域包含了1个环指结构(RING finger)和5个锌指结构，其中RING指域是许多泛素E3连接酶共有部分，具有泛素E3连接酶活性，可以催化自身生成赖氨酸63(K63)-多泛素链，介导其他下游信号分子的活化，从而诱导NF-қB组成性活化^[1]^。

近期的一项研究引起我们重视，该研究应用基于比较基因组杂交芯片技术、*Affymetrix*基因表达谱芯片技术，在346例肺癌样本中发现*TRAF6*基因扩增发生率高达34.1%，在体内外实验中，进一步证实了*TRAF6*基因扩增组成性活化NF-қB信号途径，肿瘤形成及锚着独立性生长依赖于*TRAF6*基因扩增^[2]^。

但目前*TRAF6*基因对非小细胞肺癌(non-small cell lung cancer, NSCLC)恶性生物学特征的影响作用尚未充分阐明，本研究主要针对这一方面进行进一步探索性研究。

## 1 材料与方法

### 1.1 细胞株及主要抗体

A549、H1650、Calu-3由我室传代保存，SPC-A-1细胞株购自上海细胞库，按说明书培养。CXCR4抗体购自Cell Signaling Technology公司，其余抗体(TRAF6、总泛素化抗体、β-actin、NF-қB-P65、GAPDH、MMP-1、MMP-2、MMP-9、Twist、TIMP-2、Slug、CD24、tubulin、Lamin B以及二抗)均购自Santa Cruz公司。

### 1.2 实时荧光定量聚合酶链反应(qRT-PCR检测)

Trizol法提取各实验组细胞的总RNA，紫外分光光度计准确定量。总RNA进行反转录获取cDNA后进行PCR反应。PCR反应体系以及反应条件按以往实验操作^[3]^。引物：TRAF6：正义链5’-CTATTCACCAGTTAGAGG-3’，反义链5’-GCTCACTTACATACATACT-3’；β-actin：正义链5’-TGGCACCACACCTTCTACA-3’，反义链5’-AGCACAGCCTGGATAGCA-3’。得到CT值，并按照2^-△CT^法(△CT=目的基因CT值-内参CT值)计算mRNA相对于β-actin的相对表达量进行比较^[4]^。

### 1.3 蛋白印迹(Western blot)

用RIPA裂解液裂解细胞，提取上清液。采用BCA法测量蛋白浓度后取等量蛋白质样品(20 µg/孔)，常规8% SDS-PAGE电泳，半干转膜仪转移到0.2 µM硝酸纤维素膜，5%脱脂奶粉封闭，加入一抗(1:1, 000)于4 ℃下孵育过夜，TBST清洗，HRP标记二抗(1:2, 500)室温孵育1 h，ECL显色，暗室洗片^[5]^。

### 1.4 TRAF6泛素化检测

采用蛋白印迹的方法(详见方法1.3)，检测所有发生泛素化的蛋白条带，拍照后，然后与特定蛋白的抗体和特定泛素化位点的抗体反应，显色拍照。通过阳性条带的对比来初步判断某一特定蛋白的特定位点发生了泛素化，即如果在TRAF6条带见泛素化表达，提示TRAF6很可能发生泛素化。

### 1.5 TRAF6 siRNA转染

TRAF6 siRNA购自Santa Cruz公司(TRAF6 siRNA (h): sc-36717, control siRNA (h): sc-37007)以及siRNA转染试剂盒购自(Qiagen，No.301799，美国)。收集对数期细胞，Calu-3、SPC-A-1细胞以1×10^5^个/孔接种于6孔培养板内，设TRAF6 siRNA转染组、AllStars Negative Control siRNA转染对照组以及空白对照组，并以siRNA AllStars Hs Cell Death Control siRNA转染作为监测转染效率。每组设3个复孔。在无RNA酶环境下按说明书进行操作。培养24 h及48 h后，进行后续相关检测。

### 1.6 MTS法检测细胞增殖活性

室温下静止90 min融化CellTiter 96 AQueous单溶液细胞增殖检测试剂(Promega，美国)。在96孔板中，每孔100 µL的培养液中加入20 µL的CellTiter 96 AQueous单溶液细胞增殖检测试剂，在细胞培养箱内继续孵育0.5 h。在CLD-RAD550型酶标仪检测，选取490 nm测定吸光度。设空白对照为1，检测样本与之比值即为相对细胞活性值。

### 1.7 流式细胞仪PI染色检测细胞凋亡

取对数期细胞用含10%胎牛血清细胞培养液调整细胞密度为1×10^5^接种于六孔板中，培养12 h待细胞贴壁后，转染TRAF6 siRNA，并设空白对照以及阳性siRNA转染对照组，48 h后收集细胞，PBS重悬细胞，制成单细胞，参照凋亡试剂盒(北京嘉美公司)，加入5 μL FITC Annexin V和5 μL PI避光孵育15 min后用Binding buffer洗涤，1 h内上机检测。

### 1.8 流式细胞仪细胞周期测定

取对数期细胞用含10%胎牛血清细胞培养液调整细胞密度为1×10^5^接种于六孔板中，培养12 h待细胞贴壁后，转染TRAF6 siRNA，并设空白对照以及阳性siRNA转染对照组，48 h后收集细胞，70%乙醇固定，放于4 ℃冰箱过夜。次日，用流式细胞术进行细胞周期分析。

### 1.9 凝胶迁移实验(electrophoretic mobility shift assay, EMSA)

按以往本实验室方法提取细胞核蛋白^[6]^。EMSA实验方法参照文献，探针序列为：5'-TACTAGCTACCTCGTGTCAG-3'，将6.5%非变性聚丙烯酰胺凝胶在电压为120 V条件下恒压电泳60 min，在室温下，将10 µg核蛋白加入超纯水中，30 min后再加入生物素标记探针，30 min后进行电泳100 min再印迹转移，用带正电荷尼龙膜380 mA恒流电转40 min，转膜后将尼龙膜至于紫外灯下交联15 min，再加入30m L封闭液在室温下反应20 min，将膜放入平衡液中15 min，化学发光显色，暗室洗片。

### 1.10 Transwell迁移实验

采用美国Sigma公司的铺有Matrigel基质胶的Transwell小室，Calu-3、SPC-A-1细胞以1×10^5^个/孔接种于6孔培养板内，温育24 h后转染TRAF6 siRNA，并设空白对照以及阳性siRNA转染对照组，48 h后消化离心，调整细胞密度为4×10^5^/mL，取0.2 mL接种到Transwell小室，并将Transwell小室置于24孔板内，上室内为含1%灭活血清的培养基，下室为含10%(质量分数)灭活血清的培养基。培养24 h后用棉签头擦掉小室内细胞，予90%(体积分数)甲醇固定，0.1%(质量分数)结晶紫染色，洗净风干后于荧光倒置显微镜200倍视野下拍照。实验重复3次。

### 1.11 划痕实验

经TRAF6 siRNA转染48 h后的Calu-3、SPC-A-1细胞以1×10^5^个/孔接种于6孔培养板内，并设空白对照以及阳性siRNA转染对照组，24 h后用10 µL枪头尽量垂直划痕，用PBS洗细胞3次，去除划下的细胞，加入无血清培养基，培养12 h后，显微镜200倍视野下观察拍照。

### 1.12 SPC-A-1细胞株全基因组测序

SPC-A-1细胞株经提取DNA后，送至深圳华大基因研究院进行全基因组测序，采用Agilent捕获平台进行建库，在HiSeq2000测序系统上进行全基因组测序分析(测序深度50×)。

### 1.13 统计学方法

所得数据均用SPSS 16.0软件进行数据处理。以上所有实验均至少重复3次。所有数据用均数±标准差表示，多个样本比较采用单因素方差分析，两组样本比较采用*t*检验，以*P*＜0.05表示差异有统计学意义。

## 2 结果

### 2.1 4种肺癌细胞株TRAF6表达、TRAF6蛋白K-63泛素化以及NF-қB组成性活化情况

我们选用了A549、H1650、Calu-3和SPC-A-1等4种肺癌细胞株，应用蛋白印迹和RT-PCR的方法检测TRAF6蛋白和mRNA的表达情况，在Calu-3和SPC-A-1细胞株中观察到TRAF6蛋白相对高表达(图 1A)，TRAF6 mRNA在SPC-A-1、Calu-3细胞中的相对表达量(2^-△CT^)为(0.391±0.095)、(0.365±0.075)，高于A549、H1650细胞的表达量(0.210±0.039)、(0.190±0.024)(*P*＜0.05)。TRAF6作为NF-қB通路上的关键接头蛋白，可通过自身泛素化或通过E3泛素连接酶介导其他关键信号分子的K63位点泛素化，从而激活NF-қB通路。因此，我们进一步应用蛋白印迹检测K63泛素化，发现Calu-3和SPC-A-1细胞株在TRAF6蛋白电泳位置有K63位点泛素化，该结果提示Calu-3和SPC-A-1细胞株TRAF6活化(图 1B)。另外，分别通过蛋白印迹、EMSA方法检测NF-қB-P65细胞核内表达以及NF-қB-P65与DNA结合情况，4个肺癌细胞株中，仅在SPC-A-1细胞株观察到NF-қB-P65细胞核内表达以及NF-қB-P65与DNA结合，这提示SPC-A-1细胞株NF-қB信号途径组成性活化(图 1C和图 1D)。

**1:**
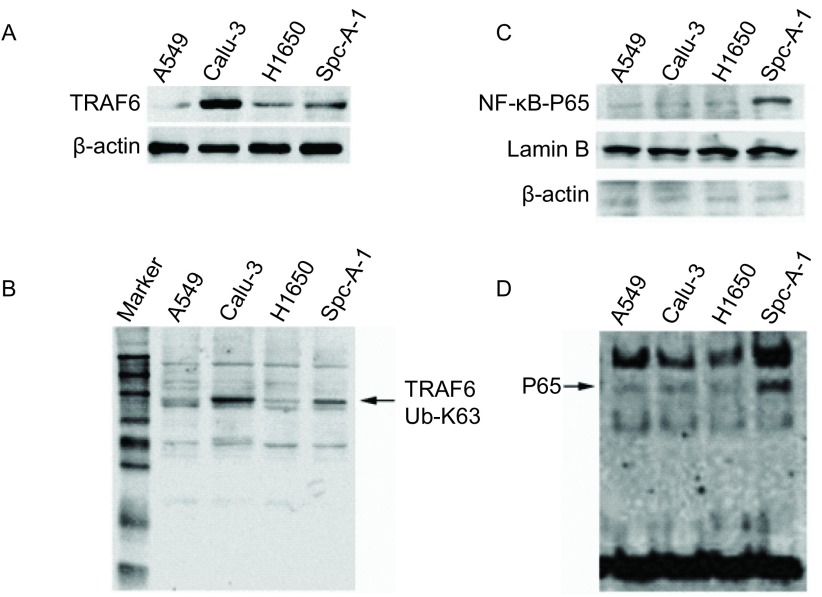
4种肺癌细胞株TRAF6表达、K-63泛素化以及NF-қB组成性活化情况。A：4种肺癌细胞株TRAF6蛋白表达。提取细胞总蛋白，以蛋白印迹方法检测TRAF6，*β*-actin作为内参。B：四种肺癌细胞株的泛素化。Calu-3和SPC-A-1细胞株在TRAF6蛋白电泳位置显示K63泛素化，提示TRAF6很可能发生泛素化。C：4种肺癌细胞株NF-қB-P65细胞核表达情况。提取细胞核蛋白进行蛋白印迹检测，以*β*-actin、Lamina作为判断细胞核蛋白提取质量及内参照。D：4种肺癌细胞株NF-қB-P65的DNA结合活性。提取细胞核蛋白进行EMSA检测。 TRAF6 expression, TRAF6 K63-ubiquitin and NF-қB constitutive activation in four lung cancer cell lines. A: TRAF6 expression of four lung cancer cell lines. Cytoplasmic lysates were analyzed by Western blot and *β*-actin was used as a loading control. B: K63-ubiquitination of TRAF6 in four lung cancer cell lines. Calu-3 and SPC-A-1 cells showed K63-ubiquitinization on the loacation of TRAF6 kDa in Western blot. C: Expression of nuclear NF-қB-P65 in four lung cancer cell lines. Nuclear lysates were analyzed by Western blot analysis. *β*-actin (cytoplasmic) and Lamin B (nuclear) were used to determine purity and as a loading control. D: DNA-binding activity of NF-қB-P65 in four lung cancer cell lines. Nuclear lysates were analyzed by EMSA. TRAF6: tumor necrosis factor receptor-associated factor 6; NF-қB: nuclear factor-*κ*B.

### 2.2 SPC-A-1细胞株全基因组测序结果

全基因组测序结果显示：SPC-A-1细胞株未发现*TRAF6*基因突变以及基因拷贝数改变，但TRAF1、TRAF2、TRAF5均发现错义突变。

### 2.3 SPC-A-1细胞株NF-κB组成性活化依赖TRAF6表达

TRAF6是NF-қB信号途径的上游关键信号分子，为了进一步明确SPC-A-1细胞株NF-қB组成性活化是否依赖TRAF6分子，我们应用TRAF6 siRNA转染SPC-A-1细胞株下调TRAF6表达，观察SPC-A-1细胞株NF-κB活性变化情况。蛋白印迹结果表明，在转染TRAF6 siRNA后48 h后，TRAF6蛋白表达受到明显的抑制，同时观察到SPC-A-1细胞株NF-қB组成性活化明显下调(图 2)。这些结果提示SPC-A-1细胞株NF-қB组成性活化依赖TRAF6表达。

**2:**
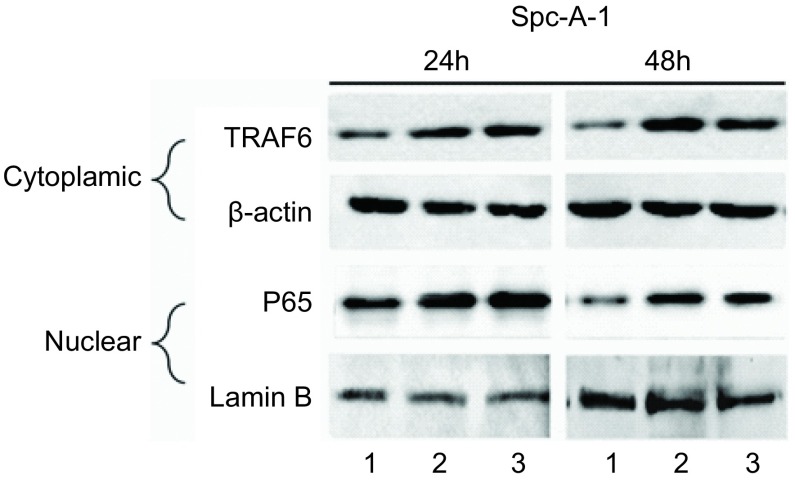
抑制TRAF6表达可以下调SPC-A-1细胞株NF-қB的活性。TRAF6 siRNA或对照siRNA转染SPC-A-1细胞株，经过24 h及48 h后，分别提取细胞浆、细胞核蛋白检测TRAF6、P65表达，*β*-actin和Lamin B作为内参照。1：TRAF6 siRNA；2：阴性对照；3：阳性对照。 Downregulation of TRAF6 reduces NF-қB activity in SPC-A-1 cells. SPC-A-1 cells were transfected by TRAF6 siRNA or control siRNA. After 24 h or 48 h, cytoplasmic lysates and nuclear lysates were analyzed for TRAF6 and P65 expression, respectively. *β*-actin and Lamina B was used as a loading control. Line 1: TRAF6 siRNA; Line 2: negative control; Line 3: control siRNA.

### 2.4 下调SPC-A-1细胞株TRAF6表达可以促进细胞凋亡，但对细胞增殖、细胞周期无明显影响

为了进一步明确TRAF6高表达对SPC-A-1细胞恶性生物学行为的潜在作用，我们通过转染TRAF6 siRNA下调SPC-A-1细胞株TRAF6表达，并应用流式细胞仪、MTS方法检测SPC-A-1细胞凋亡、细胞周期以及细胞增殖的变化。TRAF6 siRNA转染48 h后，如图 3所示，细胞的凋亡指数为(44.0%±0.98%)，与阳性对照组及阴性对照组相比，分别为(27.2%±1.86%)、(28.1%±1.45%)，差异明显(*P*＜0.05)。下调TRAF6表达并未对SPC-A-1细胞株的细胞周期、细胞增殖产生影响(资料未显示)。

**3:**
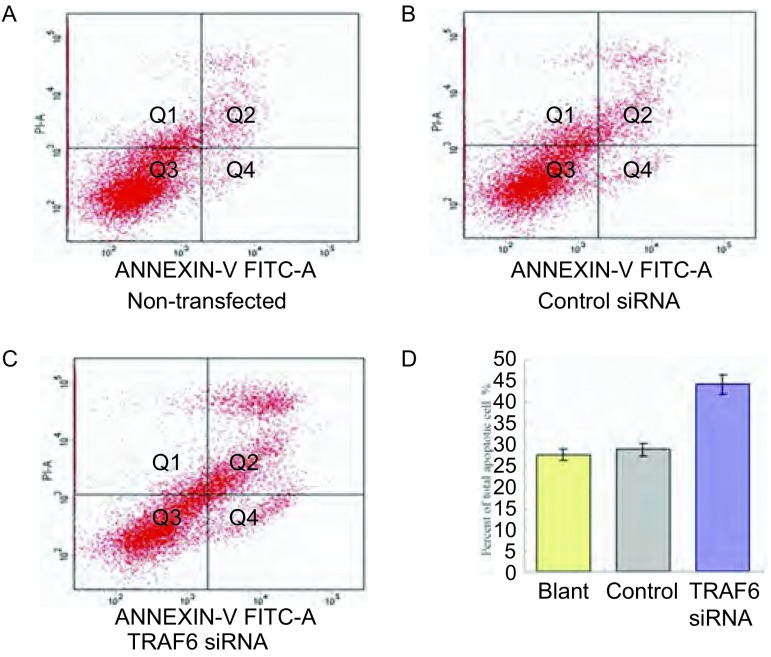
下调TRAF6表达对SPC-A-1细胞凋亡的影响。A：空白对照组；B：阳性对照组；C：TRAF6 siRNA转染组；D：凋亡细胞的相对比例。 Downregulation of TRAF6 on SPC-A-1 cell apoptosis. A: not-transfected group; B: control siRNA group; C: TRAF6 siRNA group; D: the relative content of apoptotic cells.

### 2.5 下调TRAF6表达可以抑制SPC-A-1细胞侵袭及迁移能力

我们通过Transwell小室法及划痕实验检测细胞的侵袭及迁移能力，TRAF6 siRNA转染48 h后，与空白组及对照组比较，TRAF6 siRNA转染组SPC-A-1细胞侵袭及迁移能力明显减弱(图 4)。划痕实验显示，对照组和空载缝隙分别愈合了(50.2±1.1)μm、(48.5±1.4)μm，转染组愈合了(8.3±0.7)μm，差异明显(*P*＜0.05)，说明转染TRAF6后细胞的迁移能力降低；Transwell小室实验显示，TRAF6 siRNA转染组每视野细胞数为(11.2±2.1)，与阳性对照组及阴性对照组相比，分别为(32.1±3.3)、(38.3±5.2)，差异明显(*P*＜0.05)。

**4:**
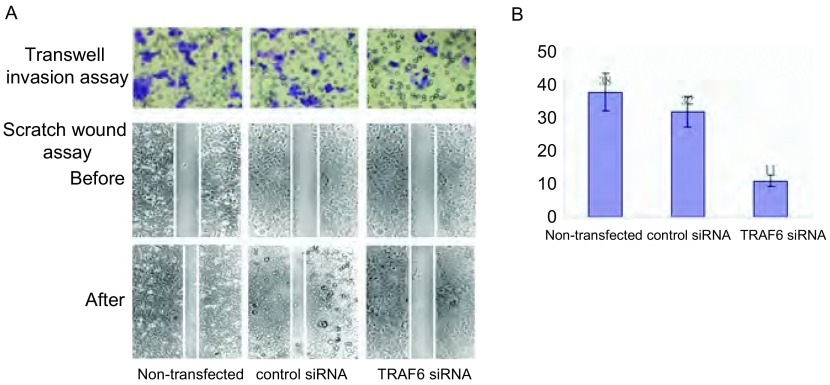
下调TRAF6表达可以抑制SPC-A-1细胞侵袭及迁移能力。A：划痕实验及Transwell小室实验；B：不同处理组SPC-A-1的侵袭细胞数。 Downregulation of TRAF6 reduces the migration and invasion capacity. A: Scratch test and transwell chamber assay; B: Number of invasive SPC-A-1 cells in the three groups.

### 2.6 下调TRAF6表达可以抑制SPC-A-1细胞CD24、CXCR4蛋白表达

为了探索下调TRAF6表达抑制SPC-A-1细胞侵袭及迁移能力的可能相关机制，我们进一步通过蛋白印迹方法检测与细胞凋亡或侵袭转移密切相关的MMP-1、MMP-2、MMP-9、Twist、TIMP-2、Slug、CD24以及CXCR4蛋白的表达。经TRAF6 siRNA转染48 h后，TRAF6 siRNA转染组与空白组、对照组相比，CD24及CXCR4蛋白表达明显下调，其余蛋白表达未见明显变化(图 5)。

**5:**
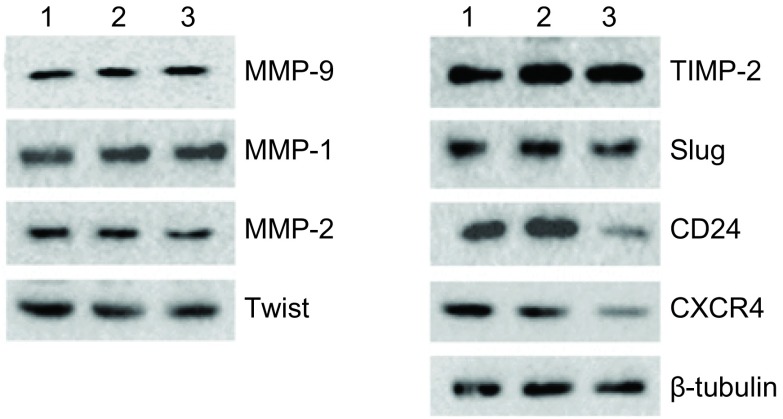
下调TRAF6表达可以抑制SPC-A-1细胞CD24、CXCR4蛋白表达。1：阴性对照；2：阳性对照；3：TRAF6 siRNA。 Downregulation of TRAF6 decrease CD24 and CXCR4 expression in SPC-A-1 cells. Line 1: negative control; Line 2: control siRNA; Line 3: TRAF6 siRNA.

### 2.7 下调TRAF6表达对Calu-3细胞株的恶性生物学的影响

我们通过TRAF6 siRNA转染Calu-3细胞株下调TRAF6表达，观察对Calu-3细胞的NF-κB活性、细胞凋亡、细胞增殖、细胞周期、迁移及侵袭能力的影响，研究结果显示TRAF6表达下调并未对Calu-3细胞株的恶性生物学产生影响(资料未显示)。

## 3 讨论

目前肺癌发病率、死亡率逐年攀升，已位居各种常见肿瘤之首^[7]^。近年来，NSCLC的靶向治疗取得了突破性的进展，因此，寻找新的切实可靠的的作用靶点是疗效突破的根本途径之一。近期研究发现*TRAF6*基因在NSCLC常见扩增，可能是NSCLC的一个驱动基因，因此本研究主要针对*TRAF6*基因在NSCLC恶性生物学表型的作用进行研究，进一步探索TRAF6作为NSCLC的潜在治疗靶点的可能性。

本研究筛选了A549、H1650、Calu-3以及SPC-A-1等4株肺癌细胞株，在4株细胞株中，Calu-3及SPC-A-1细胞TRAF6蛋白及mRNA相对高表达，而且这两株细胞株TRAF6均通过K63位点自身泛素化活化，但仅在SPC-A-1细胞株观察到NF-қB组成性活化。我们进一步通过转染TRAF6 siRNA下调TRAF6表达，观察到Spc-A-1细胞株NF-қB活性下调、促进细胞凋亡以及细胞侵袭能力明显降低，这些研究结果提示TRAF6可能通过NF-қB途径参与肺癌的凋亡、侵袭转移。一些相关文献也提示TRAF6在肿瘤发生发展过程中扮演了重要的角色，我国学者孙恒等^[8]^发现TRAF6可以结合低氧诱导因子-1α(hypoxia inducible factor 1α, HIF-1α)蛋白并对其进行K63连接的多聚泛素化修饰，阻止了HIF-1α的降解，导致该蛋白在细胞内累积，从而促进肿瘤血管的生成和肿瘤生长。另外，在不同肿瘤的研究中，研究显示TRAF6可促进肿瘤增殖^[9]^、侵袭转移^[10, 11]^、抑制细胞凋亡^[12]^。

为了进一步探索下调TRAF6促进SPC-A-1细胞凋亡以及降低细胞迁移侵袭能力的相关作用机制，我们筛查了MMP-1、MMP-2、MMP-9、Twist、TIMP-2、Slug、CD24以及CXCR4等蛋白的表达(这些蛋白均为细胞凋亡或细胞迁移侵袭中的关键分子)，发现下调TRAF6表达可明显降低CD24及CXCR4蛋白表达。一些研究表明NF-қB信号途径可以通过参与或调节CD24和CXCR4的表达促进肿瘤细胞凋亡^[13-15]^，因此，下调TRAF6表达有可能通过NF-κB-CD24/CXCR4信号轴对细胞凋亡产生影响，但仍需进一步证实，相关的具体作用机制仍有待进一步明确。

TRAF6在SPC-A-1细胞凋亡、迁移侵袭转移潜能上具有重要作用，但TRAF6是否是这些环节的关键核心节点尚有待进一步深入研究。大量的基础及临床研究表明：一个可靠的肿瘤治疗靶点至少应具备以下两个特点：①该靶点分子往往在基因水平上发生异常改变，如基因突变、扩增、异位重排或者表观遗传学改变等，导致该分子表现出异常的生物学功能；②该分子功能异常往往又是维持肿瘤恶性生物学行为的关键^[16, 17]^。但SPC-A-1细胞测序结果并未发现TRAF6发现突变或拷贝数改变，TRAF6基因在SPC-A-1细胞株中是否存在表观遗传学调控的改变尚不可知，另外，由于细胞凋亡、侵袭转移过程的信号传导途径错综复杂，TRAF6作为这个网络中的成员是否为核心作用节点仍有待更深入的研究加以阐明。

另外，本实验在Calu-3细胞株中观察到TRAF6泛素化，虽然程度高于SPC-A-1，但下调TRAF6表达对Calu-3细胞增殖、凋亡、细胞周期、迁移及侵袭能力等均未见明显影响，这提示TRAF6可能在维持细胞恶性表型中并非占据主要作用，这一不同结果可能与细胞信号途径网络背景的复杂性有关，有待于进一步深入研究。
